# General Registration of Diseases

**Published:** 1837-07

**Authors:** 


### GENERAL REGISTRATION OF DISEASES.

[We have much pleasure in giving a place in our journal to the following document, the publication of which does great credit to the authorities from whom it comes. We think there can be but one opinion among all intelligent members of the profession, of the very great importance of the object sought to be attained by the official registration of fatal diseases; and we trust that every physician and surgeon in the kingdom will be found ready and willing to contribute his aid towards its accomplishment.]

We, the undersigned, President of the Royal College of Physicians, President of the Royal College of Surgeons, and Master of the Worshipful Society of Apothecaries, having authority from the several bodies whom we represent, do resolve to fulfil the intentions of the Legislature in procuring a better registration of the causes of death, being convinced that such an improved registration cannot fail to lead to a more accurate statistical account of the prevalence of particular diseases from time to time.

We pledge ourselves, therefore, to give in every instance which may fall under our care, an authentic name of the fatal disease.

And we entreat all authorized practitioners throughout the country to follow our example, and adopt the same practice, and so assist in establishing a better registration in future throughout England; for which purpose we invite them to attend to the subjoined explanatory statement, in which they will set forth the provisions of the recent statute, and the means whereby the important object we have recommended may most effectually be attained.

(Signed)

Henry Halford, *President of the Royal College of Physicians.*
Astley Cooper, *President of the Royal College of Surgeons.*
J. Hingeston, *Master of the Society of Apothecaries.*

### *Explanatory Statement.*

The recent Act for registering births, deaths, and marriages in England, presents an opportunity for obtaining that great desideratum in medical statistics—a more exact statement of the causes of death, in the case of every registered death throughout the whole of England and Wales, after the month of June next ensuing.

The register-books in which all deaths are to be registered after the last day of June, 1837, contain columns wherein may be inserted the cause of death, in juxta-position with those other important illustrative circumstances, the sex, the age, and the profession, or calling, of the deceased person. Each register-book will also be assigned to a particular district of small extent, and will thus shew in what part of the kingdom each death has occurred. If, therefore, the cause of death be correctly inserted, there will exist thenceforward public documents, from whence may be derived a more accurate knowledge, not only of the comparative prevalence of various mortal diseases, as regards the whole of England and Wales, but also of the localities in which they respectively prevail, and the sex, age, and condition of life, which each principally affects.

For the attainment of this object it is necessary to ensure, as far as it is possible, the "cause of death." It is obvious that on this subject the requisite information can seldom be given to the registrar, except by the medical attendant on the deceased person, and that even if the registrar be a medical practitioner (which in many instances will be the case), yet will he often be unable to ascertain the truth in this respect, if he is to depend solely on the reports of persons ignorant of medicine, and of the names and nature of diseases; and it cannot be expected that from his own knowledge he will be able so far to correct their errors as to ensure a statement worthy of credit. The requisite information must therefore be supplied, either directly or indirectly, by the medical attendant of the deceased person; that is to say, if such medical attendant is not applied to by the registrar, he must afford the requisite information to those other persons to whom the registrar must apply.

The persons who, according to the act for registering births, deaths, and marriages in England, must give information to the registrar on being requested so to do, are

"some person present at the death, or in attendance during the last illness," or "in case of the death, illness, inability, or default of all such persons, the occupier of the house or tenement, or (if the occupier be the person who shall have died) some inmate of the house or tenement in which such death shall have happened." It is also provided, that "for the purposes of this act, the master or keeper of every gaol, prison, or house of correction, or workhouse, hospital, or lunatic asylum, or public or charitable institution, shall be deemed the occupier thereof."

It is therefore earnestly recommended that every practising member of any branch of the medical profession who may have been present at the death, or in attendance during the last illness, of any person, shall immediately after such death place in the hands of such other persons as were in attendance, of the occupier of the house in which the death occurred, and of some inmate who may probably be required to give information, written statements of the cause of death, which such persons may show to the registrar, and give as their information on that subject.

It is desirable that such statement should be very short, the column in the register-book in which it is to be inserted being not more than sufficient for the insertion of about ten words of moderate length. It should, therefore, contain only the name of the disease which was considered to be the cause of death, and not a detailed account either of antecedent symptoms, or of the appearances which may have presented themselves after death. It is also desirable that such statement should exhibit the popular or common name of the disease, in preference to such as is known only to medical men, whenever the popular name will denote the cause of death with sufficient precision.

---

